# Harm reduction policy in Taiwan: toward a comprehensive understanding of its making and effects

**DOI:** 10.1186/s12954-016-0101-6

**Published:** 2016-04-04

**Authors:** Jia-shin Chen

**Affiliations:** Institute of Science, Technology and Society, National Yang-Ming University, 155, Sec. 2, Linong St., Beitou District, Taipei City, 11221 Taiwan

**Keywords:** Harm reduction, Taiwan, Assemblage, HIV, Transnationality

## Abstract

**Background:**

In response to the spread of HIV caused by needle sharing among injection drug users (IDUs), the Taiwan Centers for Disease Control implemented a pilot harm reduction program in 2005 that expanded nationwide in 2006. The policy led to a significant reduction in the number of HIV-positive cases among IDUs in 4 years.

**Methods:**

This article aims to provide a critical evaluation of this harm reduction policy in Taiwan. The research leading to this article included a thorough literature review and in-depth interviews with 31 active policy participants, including people working in hospitals, the academia, non-governmental organizations, community pharmacies, the legal system, and health authorities at both the central and local levels. The collected data were analyzed on the basis of situational analysis.

**Results:**

The article examines the policy success by showing how this policy was assembled and by exposing the frictions and adjustments during its formation and implementation. Inter-departmental conflicts within or without the government and the efforts to coordinate them are addressed, and the transnational dimensions of this harm reduction policy are also discussed. The article then reflects on the effects of the policy and asks where the line should be drawn between what is harm reduction and what is not.

**Conclusions:**

This case illustration reveals the complexity of understanding an assembled health policy that involves multiple participants. The article intends to render an analytic account to enable a comparison with similar policies in other countries.

## Background

Taiwan is an island country populated by approximately 23 million people with a gross domestic product per capita of $22,632 in 2014 [1]. The portion of people living below the poverty line is 1.51 % of the population [2]. Although the percentage is a disputable number, it remains the lowest in the world. A relatively stable economy in the past few decades has resulted in Taiwan facing emerging socio-economic problems, such as unequal accumulation of capital and means of production, industrial outsourcing, booms and bubbles of land and the housing market, and a fluctuating relationship with Mainland China.

After World War II, Taiwan was ceded by Japan to China’s Nationalist government, which retreated to this island after its defeat in Mainland China to the Chinese Communist Party. In the immediate postwar period, psychoactive substance use, even within medical intervention, was strictly regulated by law. As a result, illegal drug use was not a widespread social concern until the end of martial law in 1986. The end of martial law led to the beginning of a new era characterized by exuberant communication among people and organizations. Unfortunately, this development also ushered in the smuggling of illegal drugs across the Taiwan Strait in the years to follow. In the early 1990s, Taiwan witnessed a surge in amphetamine and heroin use that quickly caught the government’s attention and led to police actions. Under the Narcotics Elimination Act, a large number of people were arrested and subsequently imprisoned for drug crimes. Eventually, they comprised the majority of jail inmates. In the mid-1990s, the Taiwanese government began to wage war on drugs and discussed possible revisions to applicable laws and policies to respond to the increase in drug crimes. Drug users were encouraged to seek adequate treatment from medical institutions before they were arrested. The revision and renaming of the Narcotics Hazard Prevention Act in 1998 ushered in a new stage, in which more participants in drug use treatment were allowed and a number of psychiatrists who specialized in addiction intervention and treatment were required. However, compared with the mainstream psychiatric community, these psychiatrists were few and marginal. Given the long-held belief that addiction is an intractable personal moral defect and the limited resources allocated to illegal drug use and users, psychiatric treatments and interventions were not fully recognized and utilized.

**Fig. 1:**
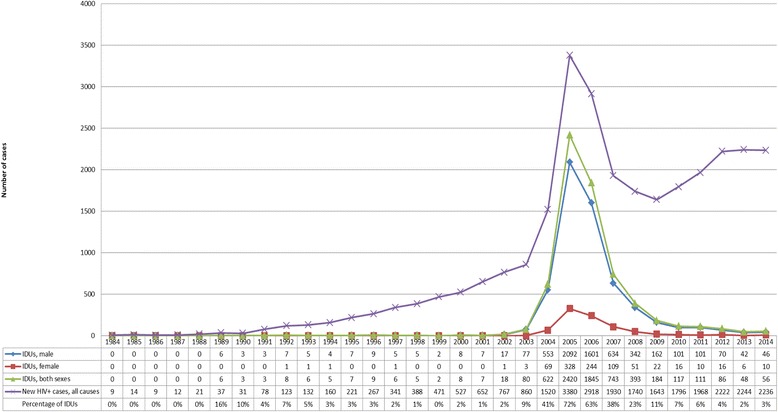
Reported number of HIV-positive injection drug users, 1984-2014. Note that the new HIV cases from all causes (*purple line*) increase in 2009 while the number representing new HIV-positive drug users (*green line*) keeps declining (adopted from TCDC [22])

Ironically, drug users, especially those who inject heroin, finally obtained their long-deserved attention from the government not because they were de-stigmatized but because they were doubly stigmatized, both as abject drug users and as dangerous public threats carrying and transmitting HIV. The first AIDS patient was identified in 1984. The Taiwan Centers for Disease Control (TCDC) was the agency responsible for the prevention and intervention of the disease. Free medical treatments for HIV/AIDS were offered by the government to ensure better control of this endemic [3, 4]. Men having sex with men (MSM) were considered the major reason for HIV infection, followed by heterosexual contact [5]. By contrast, injection drug users (IDUs), who mostly depend on heroin, were not considered as a major risk population until around 2003 (Fig. 1). The transmission of HIV among IDUs rapidly escalated in the next 2 years, eventually comprising 72 % of new cases in 2005. Sharing needles and diluting solution appeared to be the most likely route of disease transmission among this group [6]. More alarmingly, the IDU group was less likely to seek medical treatment than the sexual contact group, and this situation meant that the HIV endemic could escape medical and administrative attention more easily [5]. Thus, in response to this emerging public health threat, the TCDC announced that it would implement a pilot harm reduction program in 2005 that focused on four administrative regions (Taipei City, Taipei County, Taoyuan County, and Tainan County). These regions were selected either due to their high rates of drug use or due to their socio-political significance. The TCDC expanded the program nationwide in 2006 [5, 7].

In 4 years, the policy has accomplished a significant reduction in the number of reported cases of HIV/AIDS among IDUs (Fig. 1, see year 2005 onward). Studies have also shown that criminal arrests involving Schedule I substances (predominantly heroin) decreased over the years [8].

This article aims to provide a critical assessment of the harm reduction policy in Taiwan and to problematize the success of the policy by presenting its formation and exposing the frictions and adaptations during its implementation. This case illustration intends to render an analytic account to enable a comparison with similar policies in other countries.

## Methods

This article stems mainly from my doctoral research, which was conducted in the period of 2004–2009 [9]. This research has been supplemented with follow-up observations from then on. My doctoral research was a qualitative study that examined the unfolding of the harm reduction policy in Taiwan as a project that both promoted public health and facilitated addiction-related scientific knowledge. The goal was pursued by tracing the inter-departmental coordination within the government, the interactions between government bureaucracy and professional experts, and the engagement and evolution of various academic disciplines involved. The research included a thorough literature review and in-depth interviews with 31 active policy participants, recruited through snowball sampling and with informed consent from 2007 to 2009, that is, during the heyday of the policy implementation. These participants were clinical workers including psychiatrists, pharmacists, and case managers (*n* = 9), academic professionals devoted to HIV and/or drug research (*n* = 6), non-governmental organization (NGO) workers (*n* = 2), legal professionals (*n* = 2), and bureaucrats from health authorities at the central and local levels (*n* = 12). Some of the participants were interviewed more than once to verify or follow up certain points. Their accounts were treated not as simple statements of truth but as situated reflections of personal experience and opinion subject to analytic scrutiny. The collected data were analyzed on the basis of situational analysis, which is a postmodern version of grounded theory proposed by Adele Clarke [10]. Situational analysis aims to sort out information through a series of coding, memo-taking, and mapping. It takes collected information not as statements of reality but as parts of situated knowledge and perspective held by participants and narrators. Therefore, situational analysis does not aim to identify a single social process that accounts for a social phenomenon but to reassemble the sophisticated social phenomenon with contestations and incongruences. It is an effective analytic tool that can powerfully illustrate the discursive cacophony in a policy arena where multiple voices counter one another.

During the analysis, particular emphasis was placed on the specific manner in which this policy was designed, formulated, and implemented. Contestations and frictions during the policy process were also explored. The transnational aspect of this policy was addressed to highlight the transfer process of the novel concept of harm reduction when it first attracted public and governmental attention in Taiwan. By delineating the contour of the policy from domestic and transnational influences, this article attempts to provide a point of reference for latecomer countries within or beyond Asia that will adopt harm reduction policy and other health promotion strategies.

## Results

### Frictions of a transnational policy assemblage

The harm reduction policy in Taiwan was never a clearly planned, top-down project from the outset. Rather, it was characterized by local emergent variations and improvisations. When a pilot program targeted at IDUs was announced by the Department of Health (DOH) in 2005, harm reduction was portrayed as a three-pronged policy that would include expanded education and screening, a needle syringe program, and an opioid maintenance treatment (OMT). However, many barriers and objections to the policy implementation soon surfaced. Whereas some measures, such as free HIV testing for all pregnant women, encountered little pressure, some measures were upsetting to the public or other branches of the government, including the distribution of free needles and syringes through public health venues and voluntary community pharmacies, and the provision of free oral methadone as a maintenance treatment for heroin to avoid injection-related health consequences. These objections could be vehement, as the liberating ideas and practices of harm reduction (i.e., the government giving free needles and maintenance medications to active drug users) were simply unacceptable given the long tradition of suppression and prohibition in terms of illegal drug use. Thus, practical barriers included socio-cultural aspects and bureaucratic coordination and policy details that involved both central and local governments, NGOs, professions concerned, and drug users.

In consideration of these difficulties, the year-long pilot program pinpointed four administrative regions as previously stated. As the success of the pilot program was a primary concern, the DOH had to persuade other central government departments to work together, particularly the Ministry of Justice (representing the prosecutors) and the National Policy Agency under the Ministry of Interiors (representing the policemen). Despite the current Narcotics Hazard Prevention Act allowing for more medical intervention, the use of illegal drugs such as heroin was still viewed by most prosecutors and police officers as an act of crime, not a manifestation of an illness. A high-ranking DOH officer described the sources of the difficulty as follows:

“….First were our colleagues, especially those from the Ministry of Justice….Then, the public representatives. They usually did not agree with this idea. Third, the media. They did not like this, either. As a result, we needed to deal with these three [oppositions] all at once. How? You talked many times, many times, and it took time, a lot of time to persuade….”

Constant conflicts and negotiations over the potential effects of harm reduction practice (e.g., does it promote health or facilitate crime?) characterized the central government’s efforts to implement this policy. These disagreements also echoed the daily reality of frontline workers. A physician shared his observation on the police force in a methadone clinic:

“The National Police Agency announced that it did not encourage policemen to hang around [the clinic] and make arrests, but this order depended on the situation and it differed from time to time. When advisors from Australia came to visit us, they told us that we should work closely with the police, but not so closely that we wanted them to stay….Now we are more troubled by the inaction of the police. Drug crimes take place right here, but they simply do not intervene. Perhaps it’s because they do not get credit for it.”

Maintaining an optimal distance from the police and prosecutors seemed to be an important issue for health workers devoted to harm reduction measures, centrally and locally. This issue also mattered for people working in hospital settings and in community pharmacies that provided free needles and syringes. I addressed elsewhere [11] that the improvisation of these workers could be necessary for effective education. These improvisational efforts included a spatial arrangement (e.g., where to place the paraphernalia) that optimized the quality of information and communication.

Frictions between bureaucracies did not take place between the DOH and other departments. Rather, they existed within the DOH itself, as manifested in the choice of methadone over buprenorphine. This example is an excellent illustration of the differences in opinion between TCDC, then in charge of the harm reduction policy from an endemic control perspective, and the National Bureau of Controlled Drugs (NBCD), a DOH branch supervising the production, registration, and proper use of controlled narcotics including morphine, methadone, and other opiates. TCDC preferred methadone because of its administrative familiarity, lower cost, better known (not necessarily fewer) adverse effects, and a longer history of clinical use. By contrast, buprenorphine, especially when it is combined with naloxone (e.g., Suboxone®) to prevent intravenous use, has less addictive potential and less danger when overdosed. Thus, the NBCD advocated buprenorphine/naloxone as a better option for maintenance treatment. However, its high price and the relative lack of experience in large-scale implementation made this combination an unfavorable alternative [12]. Such frictions and their resolutions reflected the contradicting rationalities of the government. NBCD preferred the option that was less addictive and dangerous, but TCDC wanted a safe and inexpensive fix to the threat of an impending epidemic of HIV/AIDS. Eventually, the judgment call was made by the one “in the driver’s seat” (i.e., TCDC), as one respondent informed me.

The lack of coordination occurred not only among different units in the central government but also between the central and the local health authorities. A local health director described the policy as a back and forth feedback process between TCDC and the local health bureaus.

“TCDC knew of the problem, but it did not know what to do, so in the end it was we that first proposed the policy measures. They simply said yes, let’s find some other counties or cities to do this, a pilot program. A pilot program that each county or city made on its own, because TCDC did not have a better way or method that they saw fit.”

Contrary to being a well-orchestrated plan, the harm reduction program set out to be a “policy by crisis,” as another of my respondent aptly described. A “policy by crisis” in this case means the policy was expected to solve the problem at the lowest cost in the shortest time. Consequently, a loosely connected assemblage emerged, which I call the office elsewhere [12]. The office is composed of heterogeneous components and participants with varied concerns and ideologies in the processes of policy organization and implementation. The office is pivotal to policymaking and the eventual social reconfiguration brought about by the policy.

In addition to the inter-departmental coordination or the lack thereof within the government, the assemblage of harm reduction policy in Taiwan is also salient in its transnational dimension, which can be understood in terms of epidemiology, diplomacy, and practical policy know-how.

First, the findings in molecular epidemiology indicated that the HIV strain that caused the epidemic was similar to the one that originated in China [13–15]. These findings imply that a transmission route from China to Taiwan could be attributed to the increase in mutual communication over the years. A number of respondents in my interview series corroborated this speculation. For example, a senior addiction psychiatrist recalled that in the early 1990s, illegal substances such as amphetamine and heroin increased during the post-martial law period. “When more trade among Taiwan, Mainland China, and Southeastern Asia prospered, many people traveled to places like Thailand and Hong Kong, and sneaked in drugs,” he stated. The transnational spread of HIV followed the transnational trajectory of drugs and set the stage for harm reduction, which is a transnational assemblage through and through.

Second, given that contagious diseases, such as HIV/AIDS and severe acute respiratory syndrome, do not recognize national borders, participation of and coordination through international health communities are particularly indispensable. The recently proposed One Health concept attests to the importance of international coordination, particularly in light of infectious diseases such as HIV and globally transposable addictive substances. However, given that Taiwan is not a member country of the United Nations and World Health Organization, the country is isolated in this respect. This awkward political situation often baffles enthusiastic technocrats. An upset, middle-rank health officer stated the following:

“When WHO becomes a political organization, it becomes relatively unfair to Taiwanese people. Why should we be treated like this? Somewhat like a colonized land but worse than the status of a colony. A colonized land at least has a ruling master, but we have nothing. We simply lack an identity to participate…to gain a vision for Taiwan and connect with the international community. Our director hopes our [harm reduction] program can achieve that goal. Anti-TB or anti-AIDS, it does not matter.”

Although I do not entirely agree with his viewpoint, particularly on the part of being worse than a colony, I do sympathize with his pain and helplessness as a policy organizer. He also raised a point that this harm reduction policy, if done well, can be a useful leverage for Taiwan to acquire international recognition. This ambition, although not overtly spelled out, appears to be shared by many contributors of this policy. Thus, a domestic policy such as harm reduction always aims for global recognition.

Third, the early stage of Taiwan’s harm reduction policy was characterized by local wisdom and experience crystalized in the proposed plan, as well as by foreign experts and suggestions that guided health organizers and workers through policy implementation. Policy designs and experiences were also appropriated selectively on the basis of informal connections, such as personal recommendation and familiarity. Although the USA has often been used as an example, influences from Australia and Hong Kong were more valued during the strategy design. A former TCDC officer recalled her business trip to Australia, where she encountered Dr. Alex Wodak:

“He was very enthusiastic, and he scheduled our trip to NGOs, religion-based detox programs, safe injection rooms, and sexually transmitted diseases prevention stations in the red light district. Everything. A whole package. When we returned, we invited a professor from the University of North Wales (sic) to train our people to implement the pilot program of harm reduction.”

Considering the lack of formal connection with and official assistance from the international community, the knowledge and information comprising the policy came from the online sources of international organizations such as UNAIDS and UNODC and through international conferences, workshops, educational tours, and talks of invited foreign experts. These materials, including treatment guidelines, self-help guidebooks, and even forms for clinical or administrative use, were either translated into Chinese or revised for local use. Hence, the materials were assembled but were not well-organized. These efforts demonstrated a two-way process wherein central and local governments reciprocated and government and NGOs worked in parallel. Despite their shared commonalities, these efforts through informal and meandering channels were illustrative of the transfer process of the policy, which is distinct from that of other countries sponsored by UNODC, WHO, or regional HIV/AIDS organizations such as Asian Harm Reduction Network [16, 17].

### Policy success and its discontents

In four short years, the severity of HIV infection among IDUs dropped drastically and MSM once again became the major risk factor (Fig. 1). Thus, the harm reduction policy of Taiwan was showcased by TCDC as a success in public health administration. In 2009, TCDC decided to “routinize” the harm reduction policy, including assigning the responsibility of supervising methadone maintenance treatment to the Division of Medical Affair because this treatment became a regular medical practice and not an emergent measure against endemics. Thereafter, the central government underwent a series of re-structuring over the next few years. In 2013, the new Ministry of Health and Welfare replaced the old DOH in terms of health policies and a unit under its Division of Mental and Oral Health was exclusively devoted to substance use. According to available information on the website of the Ministry of Health and Welfare, there were 108 certified hospitals for substitutive treatment and 54 medicine-distributing sites (usually health stations and local clinics) in December 2015. In February 2016, there were 837 distribution sites for clean needles and syringes, 417 vendor machines selling clean needles and syringes, and 651 sites for recycling used paraphernalia [18, 19]. At first glance, the implementation of this policy seems to have been successful. However, some questions remain unsolved.

A successful policy achieves its goal. In this case, the policy goal of harm reduction is to decrease the number of HIV-positive drug users, as stated in the monthly reports of TCDC. However, the decline of newfound HIV-infected IDUs actually occurred (around July 2005) prior to the implementation of the harm reduction policy on the ground [8, 20]. In that case, is it still valid to say that the policy is successful? Which part of the policy is the most effective and warrants more resources?

This decline-before-policy issue has been addressed and explained by several authors [7–9, 20]. These explanations point to the effect of large-scale screening prior to the policy implementation [7], highlight the importance of NGOs in promulgating health information among vulnerable populations [8, 21], or conceive of the policy as a platform for building citizen addicts by realigning responsibilities and entitlements [20]. These explanations may stand at the same time, but altogether, they uncover some pivotal issues concerning the evaluation of this policy: When does the policy start in terms of its effects? What and who should be included in the assessment, and for what? How should this policy be evaluated?

My intention here is to point out that, in a sociological sense, these questions concern the range of governance for HIV/AIDS and drug use. For the sake of clarity, a nuanced difference has to be delineated between government and governance: the former often refers to the administrative institutions and policy decisions that execute the plan of governing, whereas the latter tends to address the collective understanding and behavioral orientations that constitute the activities and effects of governing. When the focus shifts from policy to governance, the exact date of policy implementation will no longer be important. At this point, what really matters is the insidious and gradual formation of a collective understanding, along with its behavioral consequences, that certain health ideals (e.g., not sharing diluting solution and contaminated needles) should be encouraged and reinforced through a series of governmental strategies.

A seasoned psychiatrist, one of the first harm reduction practitioners in Taiwan, described the method of information transmission among IDUs. He stated that in clinical settings, “[IDUs] do not seem to read newspapers often; they do not watch TV often, either….But the power of word of mouth is tremendous among them….” Later during the interview, he further explained why methadone maintenance treatment alone cannot account for the effects of harm reduction: “Methadone coverage is comparatively low. It’s less than 10,000 persons now, but it is estimated that there are 60,000 to 80,000 heroin users all over the country.”

The power of word of mouth is generally believed to have existed among this clandestine group of IDUs long before the pilot program. Thus, the discussions, deliberations, and formulations before the formal implementation of a policy may facilitate the transmission of health information and generate the health outcomes anticipated by the policy. The assessment of a policy’s success should not focus merely on governmental efforts after a policy is announced and implemented. Sometimes, a policy becomes active before it is even announced to be active; hence, more participants should be taken into account, especially those who are covert or implicated. NGOs are certainly pivotal contributors [8], but people who would not otherwise be considered as educators, such as parole officers and prosecutors, also play a role in distributing information and facilitating communication. In other words, information, education, and communication in the notion of drug and HIV education must be understood in a broader perspective [11].

For example, deferred prosecution is a semi-compulsory strategy, in which arrested drug users are mandated to participate in an OMT in exchange for a withheld prosecutorial process. A prosecutor who contributed to this measure associated his motives with previous collaborations with environmental groups to manage illegal garbage disposal and community life camps, in which prosecutors worked with school educators. In these camps, prosecutors encountered problematic youths from various educational institutions and attempted to redirect their interests and energy by way of group activities. Through these interventions, enthused prosecutors not only forestalled the possibility of disruptive behavior but also familiarized themselves with the social reality of crimes and criminals.

“This practice makes prosecutors more than the people in power that deal with [criminal] cases. They may care for many things and open their minds. They will pay attention to how to tidy up their communities….Harm reduction is something similar that we do.”

In my study, many―if not all―prosecutors and parole officers shifted among the roles of punishment, surveillance, and education. However informal, unrecognized, and non-certified, they are indeed health educators in a pragmatic sense [11].

These considerations lead us back to the last question: How should we evaluate the policy? Considering that the implemented policy is part of the harm reduction governance, an adequate evaluation must first problematize the extent of the policy rather than take it for granted. Moreover, instead of viewing the policy as a three-pronged program, as claimed by TCDC, a comprehensive evaluation necessitates that this policy should be understood as being embedded in a large number of participants and actions that collectively constitute the eventual contour of measures categorized as harm reduction. Thus, a more pertinent question would be “Where do we draw the line between what is harm reduction and what is not?”

## Discussion

This article provides a sociologically informed analysis of the harm reduction policy and addresses several issues specifically observed in Taiwan but may also be shared by other Asian countries. Without sugarcoating the policy process, this article demonstrates the ways in which various participants, regulations, and institutions were assembled internally and externally to make the harm reduction policy work. By doing so, this article aims to problematize the claimed policy success and endeavors to suggest that the key factor may lie in the facilitation of communication among a multiplicity of stakeholders, planners, participants, and policy targets beyond the usually defined policy scope. This perspective can be evidenced by the fact that some mistakes in the early phase of policy implementation (such as distributing syringes of wrong sizes and wrapping up health information in oversized packages) could be attributed to the ignorance and misunderstanding of policy targets, i.e., IDUs [9]. My research repeatedly showed that an in-depth dialogue with IDUs effectively enhances the efficacy of health education by aligning the needs of IDUs with the purposes of health promotion [11].

## Conclusions

In conclusion, portraying the policy as an assembling process has several advantages that I believe will benefit our understanding of the ways in which harm reduction may develop in Asia. First, this approach will help us see the policy as a complex process with multiple directions of exchange of knowledge, personnel, and resources. The idea of an assemblage implies a heterogeneous and precarious connection that is neither easily stabilized by state action nor explained away by participants’ common goals. What my research found is an aggregate of participants with diverse professional orientations and practical concerns. Although professionals working with people with HIV temporarily formed liaison with addiction specialists, the cooperation became shaky and intermittent once the harm reduction policy in Taiwan lost momentum around 2009. These associations and disconnections, animated by policy changes, seriously affected the real everyday lives of drug users on the street. Therefore, a policy is a collective social action that involves not only the street but also the office [12], the communication and interaction between both being an empirical question that is open to further scrutiny.

Second, this article also stresses the transnational aspect of harm reduction policy, which is crucial to the rest of Asia, because harm reduction as a concept and a practice is mostly an “imported” thing for most Asian countries. Influences and directions from international organizations or non-formal channels are significant and warrant further clarification and investigation. More comparative studies will be needed to examine how this transnational perspective illuminates not only the similarities and differences but also the continuities and disjunctions between policies of different Asian countries. This type of research will help build a transnational liaison against diseases such as HIV/AIDS.

Third, the assemblage approach implies a dynamic understanding of the policy processes in Asian countries. This approach is helpful given the trend of drug use and policy reformulation. For example, Taiwanese scholars showed that drug crimes involving Schedule I controlled substances have steadily decreased over the recent years, but crimes involving Schedule II substance (mainly amphetamines) have been slowly increasing [8]. In the past few years, party drugs such as ketamine and ecstasy became a new focus of drug control and HIV/AIDS prevention in Taiwan because of their association with sexual behavior. The vicissitudes of the patterns and “fads” of drug use implies the necessity of repeatedly forming a new assemblage of harm reduction with new participants, interventions, and new medications. Distributing clean needles and syringes obviously does not work in this case. For enthusiastic harm reduction workers, the wax and wane of governmental attention on drug use and HIV/AIDS raises the importance of learning the ways of a fox rather than those of a hedgehog.

## References

[1] National Statistics, Executive Yuan, Taiwan. A summary of national income, 1951-2014. In special section on statistics. Directorate-General of Budget, Accounting and Statistics, Executive Yuan, Taiwan, 2015. http://www.stat.gov.tw/ct.asp?xItem=33338&ctNode=3565&mp=4. Accessed 6 May 2015

[2] Division of Statistics. Numbers of low-income families and persons. In: Yearly report on social welfare statistics. Ministry of Health and Welfare, Taiwan, 2015. http://www.mohw.gov.tw/cht/DOS/Statistic.aspx?f_list_no=312&fod_list_no=4177. Accessed 6 May 2015.

[3] Fang CT (2004). Decreased HIV transmission after a policy of providing free access to highly active antiretroviral therapy in Taiwan. J Infect Dis.

[4] Fang CT (2007). Cost-effectiveness of highly active antiretroviral therapy for HIV infection in Taiwan. J Formos Med Assoc.

[5] Yang CH (2008). The changing epidemiology of prevalent diagnosed HIV infections in Taiwan, 1984-2005. Int J Drug Policy.

[6] Chen CH (2008). Risky behaviors for HIV infection among male incarcerated injection drug users in Taiwan: a case-control study. AIDS Care.

[7] Lee HY (2012). Essentiality of HIV testing and education for effective HIV control in the National Pilot Harm Reduction Program: the Taiwan experience. Kaohsiung J Med Sci.

[8] Lyu SY, Su LW, Chen YMA (2011). Effects of education on harm-reduction programmes. Lancet.

[9] Chen JS (2009). Assembling harm reduction policy in Taiwan, PhD Dissertation in Sociology.

[10] Clarke AE (2005). Situational analysis: grounded theory after the postmodern turn.

[11] Chen JS (2014). Education as networking: rethinking the success of the harm reduction policy in Taiwan. Health.

[12] Chen J-S (2011). Studying up harm reduction policy: the office as an assemblage. Int J Drug Policy.

[13] Chen YM, Kuo SH (2007). HIV-1 in Taiwan. Lancet.

[14] Lin YT (2007). Molecular epidemiology of HIV-1 infection and full-length genomic analysis of circulating recombinant form 07_BC strains from injection drug users in Taiwan. J Infect Dis.

[15] Chen KT (2009). The changing face of the HIV epidemic in Taiwan: a new challenge for public health policy strategies. AIDS Patient Care STDS.

[16] Thomson N (2013). Harm reduction history, response, and current trends in Asia. J Food Drug Anal.

[17] Burnet Institute. Harm reduction in Asia: progress towards universal access to harm reduction services among people who inject drugs, United Nations Office for Drugs and Crime. 2010. https://www.unodc.org/documents/southeastasiaandpacific//2010/03/harm-reduction/UNRTF_report_2009_update_of_harm_reduction_in_Asia_FINAL.pdf Accessed 6 May 2015.

[18] Centers for Disease Control, Taiwan. Statistics of nationwide clean needles and syringes sites, vendoring machines, and recycling bins. In: Nationwide sites for clean needles and syringes. Ministry of Health and Welfare, Taiwan. 2015. http://www.cdc.gov.tw/professional/list.aspx?treeid=7B56E6F932B49B90&nowtreeid=981AAD792FA60FD4 Accessed 11 March 2016.

[19] Division of Mental and Oral Health. 102-104 List of appointed institutions for addiction treatment. In: List of appointed institutions for addiction treatment. Ministry of Health and Welfare, Taiwan. 2016. http://www.mohw.gov.tw/CHT/DOMHAOH/DM1_P.aspx?f_list_no=184&fod_list_no=5194&doc_no=3706&rn=906450796 Accessed 11 March, 2016.

[20] Chen J-S (2011). Beyond human rights and public health: citizenship issues in harm reduction. Int J Drug Policy.

[21] Morisky DE, Lyu SY, Urada LA (2009). The role of non-formal education in combating the HIV epidemic in the Philippines and Taiwan. Prospects.

[22] Centers for disease control, Taiwan. Statistics on HIV/AIDS. Ministry of Health and Welfare, Taiwan. 2015. http://www.cdc.gov.tw/info.aspx?treeid=1F07E8862BA550CF&nowtreeid=6C5EA6D932836F74&tid=5250BA9AD485D6C3 Accessed 11 March, 2016.

